# Preventive Effects of Intrauterine Injection of Bone Marrow-Derived Mesenchymal Stromal Cell-Conditioned Media on Uterine Fibrosis Immediately after Endometrial Curettage in Rabbit

**DOI:** 10.1155/2020/8849537

**Published:** 2020-11-07

**Authors:** Sanaz Bazoobandi, Nader Tanideh, Farhad Rahmanifar, Shahrokh Zare, Omid Koohi-Hosseinabadi, Iman Razeghian-Jahromi, Mehdi Dianatpour, Masoumeh Ahmadi, Arezoo Khoradmehr, Iraj Nabipour, Zahra Khodabandeh, Amin Tamadon

**Affiliations:** ^1^Stem Cells Technology Research Center, Shiraz University of Medical Sciences, Shiraz, Iran; ^2^Department of Pharmacology, Medical School, Shiraz University of Medical Sciences, Shiraz, Iran; ^3^Department of Basic Sciences, School of Veterinary Medicine, Shiraz University, Shiraz, Iran; ^4^Laparoscopy Research Center, Shiraz University of Medical Sciences, Shiraz, Iran; ^5^Central Research Laboratory, Shiraz University of Medical Sciences, Shiraz, Iran; ^6^Cardiovascular Research Center, Shiraz University of Medical Sciences, Shiraz, Iran; ^7^The Persian Gulf Marine Biotechnology Research Center, The Persian Gulf Biomedical Sciences Research Institute, Bushehr University of Medical Sciences, Bushehr, Iran

## Abstract

Uterine fibrosis is an acquired disorder leading to menstrual irregularities, implantation impairment, and abortion. Mesenchymal stromal cells (MSCs) have antifibrotic properties through chemokine secretion. MSC-conditioned media (MSC-CM) contain paracrine components—exosomes—with a great potential for repairing damaged tissue or preventing fibrosis. The main goal of this study was to evaluate the preventive effects of bone marrow-derived MSC-CM (BM-MSC-CM) on uterine fibrosis after uterine curettage in rabbits. This study included 12 female rabbits (24 uterine horns in total). Excised uteri of each of the 12 female rabbits were randomly divided into four groups of intact negative control, curettage positive control, BM-MSC injection, and BM-MSC-CM injection in the way that two corresponding uteri from a rabbit were allocated to different groups. The MSC-CM were collected from cultivated BM-MSCs 48 hours after having been washed three times and replaced in serum-free media. Through a surgical approach, the caudal parts of the uteri were submitted to traumatic endometrial curettage, except for the intact negative uteri. After suturing the uterine walls, BM-MSCs or BM-MSC-CM were injected in the curettage site. Endometrial regeneration was histologically evaluated 30 days after treatment. Based on the evaluation of histomorphometric indices, curettage with or without preventive injections increased the growth of endometrial layers. However, the amount of fibrotic tissue in the CM and the BM-MSC injection groups was the same as the normal control groups, and all were less than the curettage group. A single injection of CM of MSCs after 30 days prevented the fibrotic tissue formation induced by curettage in endometrial layers of rabbits. Injecting BM-MSC-CM immediately after curettage prevented and reduced the uterine fibrosis similar to BM-MSCs in a rabbit model.

## 1. Introduction

Uterine fibrosis is caused by the destruction of the endometrium and partial to complete closure of the uterus, which leads to infertility, recurrent pregnancy loss, and menstrual abnormalities after endometritis or aggressive curettage [1]. Intrauterine adhesions can develop from endometrial basal layer lesions caused by curettage of the newly pregnant uterus, endometriosis, abortion, and uterine tuberculosis. The syndrome may also occur after hysteroscopic operations such as myomectomy, polypectomy, endometrial ablation, or uterine artery embolization. Symptoms of uterine fibrosis include amenorrhea, abortion, and infertility. Several methods have been developed for the treatment of uterine fibrosis, with surgical intervention using hysteroscopy to remove the adherent tissue inside the uterine cavity being the first option [2]. Recently, mesenchymal stromal/stem cell therapy [3] and mesenchymal stromal/stem cell-derived macrovesicle injection [4, 5] have been recommended for the treatment of uterine fibrosis. There is no modality to prevent uterine fibrosis after curettage.

Mesenchymal stromal/stem cell therapy and its derivatives play an important role in medical and pharmaceutical fields. Among the many types of mesenchymal stromal cells (MSCs), bone marrow-derived MSCs (BM-MSCs) are multipotent stromal cells capable of differentiating into diverse somatic cells [6, 7]. In recent years, the significance of BM-MSCs in the treatment of uterine fibrosis has gained scientific attention and its application has been extensively reported in animal models [8, 9] as well as in humans [10]. Clinical trials have implicated intrauterine transplantations of bone marrow, umbilical cord, adipose tissue, and menstrual-derived stem cells as beneficial for the regeneration of the endometrium [11].

After transplantation, BM-MSCs can migrate and accumulate in endometrial tissue [9]. It is commonly approved that transplanted cells can provide morphological and functional benefits through multiple mechanisms including, but not restricted to, trophic support, cell replacement, regeneration of endogenous cells, immunosuppression/anti-inflammation, stimulation, and regulatory interactions with endogenous cells [9]. Hence, the BM-MSCs might have an important role in endometrial rebuilding, at least in terms of cellular exchange and inflammatory stimuli [12]. Mesenchymal stromal/stem cell therapy for the regeneration of the endometrium has proven to be a great promise for repair and regeneration of the injured tissues [13].

However, since impractical simultaneous preparation and administration of mesenchymal stromal/stem cells hinder their application in the treatment of uterine fibrosis when uterine injuries occur, the provision of an alternative approach to the direct use of stromal/stem cells sounds necessary. Accumulative evidence suggests the replacement of stromal/stem cells with secreting MSC microvesicles in the treatment of uterine fibrosis [4, 5]. Accordingly, administration of the stromal cell culture medium containing microvesicles for the prevention of uterine fibrosis concurrent with uterine curettage might prove a favorable substitute to mesenchymal stromal/stem cell therapy. This study used bone marrow-derived mesenchymal stromal cell- (BM-MSC-) conditioned media (CM) in a rabbit model suffering from uterine fibrosis, and the results were compared with those of BM-MSC injection.

## 2. Methods

### 2.1. Ethical Approval Statements

This investigation was performed in accordance with relevant guidelines and regulations on animal studies, and all experimental protocols were approved by the ethical committee of Bushehr University of Medical Sciences (Permission number: IR.BPUMS.REC.1399.084).

### 2.2. Animals

Thirteen healthy adult female (1 donor and 12 recipients) New Zealand white albino rabbits (*Oryctolagus cuniculus*), weighing between 3500 g and 4000 g were purchased from and housed in the Center of Comparative and Experimental Medicine, Shiraz University of Medical Sciences. They were maintained individually in stainless steel cages under appropriate conditions (temperature: 20 ± 2°C; humidity: 60%; 12 h light/dark cycle) with free access to food and water. Their food was supplemented by adding carrot and parsley.

### 2.3. BM-MSC Isolation

To establish the BM-MSC culture, both femur and tibia from a rabbit were excised and carefully cleaned of adherent soft tissue under sterile conditions. The ends of the bones were cut away, and bone marrow was harvested by flushing with Dulbecco's Modified Eagle's Medium (DMEM) supplemented with 10% fetal bovine serum (FBS), 1% penicillin and streptomycin (Sigma-Aldrich), and 1% L-glutamine (Sigma-Aldrich), with the use of a 10 mL syringe. After washing and undergoing centrifugation at 1200 rpm for 5 min, the cell pellet was resuspended in fresh medium and cultured in supplemented DMEM medium inside a 75 cm^2^ flask. The flask was incubated in a CO_2_ incubator (5% CO_2_, 37°C, and saturated humidity). The first culture medium was changed after 24 h to remove nonadherent cells.

The adherent cells were cultured till 80-90% confluence undergoing medium exchange every 3-4 days and then passaging to expand the MSC population. The adherent cells were washed with phosphate-buffered saline (PBS), and the cells were harvested after 2-3 min treatment with 0.25% trypsin (Gibco). Then, the enzyme was inactivated with the same amount of culture medium. Spindle-shaped morphology of BM-MSCs was observed using light microscopy at every passage round. Two subcultures were performed taking about a week in order to provide a sufficient cell yield for optimal evaluation of cell-specific characteristics required for cell therapy. Cells in the second passage were collected and counted using a hemocytometer.

They were cryopreserved through the conventional method by dimethyl sulfoxide (DMSO; MP Bio, France) and were aliquoted into sterile cryovials at a density of 2 × 10^6^ viable cells/mL. Before cell characterization or cell therapy, the frozen cryovials were quickly thawed in a 37°C water bath. Before the ice clump was completely thawed, 1 mL of supplemented DMEM medium was added. After resuspension of the cells in the fresh medium, they were cultured and subcultured just once in the same condition and medium as explained above. Besides morphology and plastic adherent features of isolated cells, they also were examined for their potential differentiation into adipocytes and osteoblasts along with surface markers to confirm their characteristics.

### 2.4. Reverse Transcription-Polymerase Chain Reaction (RT-PCR)

BM-MSCs were examined for the expression of surface markers using RT-PCR. Total RNA of BM-MSCs at passage 3 was extracted according to manufacturer's instructions using a Column RNA Isolation Kit (DENAzist-Asia, Iran). Total RNA concentration was determined by nanodrop spectrophotometry. Before reverse transcription, the RNA samples were digested with DNase to remove contaminating genomic DNA. After that, complementary DNA (cDNA) synthesis from DNA-free RNA (500 ng) samples was done using the AccuPower CycleScript RT PreMix Kit (Bioneer, Korea) according to the manufacturer's protocol.

Specific primers were designed based on sequences corresponding to highly conserved regions of CD34, CD45, and CD73 in rabbits. The primer sequences used are summarized in Table 1. The microtubes containing the requirements of a PCR reaction up in a 20 *μ*L mixture were transferred to a Thermocycler (Eppendorf Mastercycler Gradient, Eppendorf, Hamburg, Germany). The RT-PCR amplification conditions for the surface markers were performed in 30 cycles of amplification including denaturation at 95°C for 30 sec, annealing at 64°C for 30 sec, and extension at 72°C for 30 sec, with a deployment of 2 min at 95°C for primary denaturation and 5 min at 72°C for the final extension. PCR products were run on 1.5% agarose gel electrophoresis and visualized by UV light (UVITEC, Cambridge, UK).

### 2.5. Osteogenic and Adipogenic Differentiation Assay

In order to evaluate the differentiation potential of BM-MSCs, cells at passage 3 were used and osteogenic differentiation and adipogenic differentiation were induced. For osteogenic differentiation, BM-MSCs were seeded in a 6-well plate. After the cells reached 70% confluence, they were cultured for 3 weeks in osteogenic medium containing low glucose DMEM supplemented with 100 nM dexamethasone (Sigma-Aldrich), 0.05 mM ascorbate-2-phosphate (Wako Chemicals, Richmond, VA, USA), 10 mM b-glycerophosphate (Sigma-Aldrich), 1% antibiotic/antimycotic, and 10% FBS. Half of the medium was replaced every 3 days. At day 21, the cells were fixed by a 10% formalin solution (Sigma-Aldrich) and then stained using Alizarin red (Sigma-Aldrich) to detect calcified extracellular matrix and osteogenic differentiation.

For adipogenic differentiation, BM-MSCs were seeded in a 6-well plate. After reaching 70% confluency, adipogenic differentiation was induced with adipogenic induction medium containing DMEM low glucose, 10% FBS, 0.5 mM isobutyl-methylxanthine (Sigma-Aldrich), 10% FBS, 0.5 mM isobutyl-methylxanthine (Sigma-Aldrich), 1 *μ*M dexamethasone, 10 *μ*M insulin, and 200 *μ*M indomethacin (Sigma-Aldrich). The plates were maintained for three weeks and medium was replaced every 3-4 days. At the end of the period, cultures were fixed by 10% formalin solution for 10 minutes. Fixed cells were subjected to oil red O (Sigma-Aldrich), which specifically stains lipid droplets.

### 2.6. Cell Counting, Growth Curve, and Calculation of Population-Doubling Time (PDT)

Growth curves were plotted for BM-MSCs derived from rabbit bone marrow in order to evaluate growth kinetics of the cells. For the assessment of growth characteristics, BM-MSCs at passage 3 were seeded in a 24-well plate at a density of approximately 7.5 × 10^4^ cells per well in triplicate. Cells were collected from each well 1–7 days after seeding and counted microscopically to draw a cell growth curve. The curve was drawn using GraphPad Prism (version 5.01; GraphPad Software Inc., San Diego, CA, USA).

To evaluate the in vitro proliferation rate, the PDT value was determined for each studied cell type. PDT was calculated using the formula PDT = *T*ln2/ln(Xe/Xb), where *T* is the incubation time in hours, Xb represents the cell number at the beginning of the incubation time, and Xe corresponds to the cell number at the end of the incubation time.

### 2.7. Preparation of BM-MSC-CM

The MSC-CM were collected from the third passages of cultivated BM-MSCs. In order to obtain the CM, BM-MSCs at passage three were cultured at a density of 10^6^ cells in a T75 flask. At 80 to 90% confluence, the BM-MSCs were washed three times with PBS, and the media were replaced with 10 mL of FBS-free DMEM. After 48 h incubation, the media were collected and filtrated through a 0.2 *μ*m filter to remove cellular debris and stored at -80°C until use.

### 2.8. Surgical Procedure

For induction of the model and treatment, double uteri of each of the 12 female rabbits were randomly divided into four groups of intact negative control, curettage positive control, BM-MSC injection, and BM-MSC-CM injection in the way that two corresponding uteri from a rabbit were assigned into different groups (*n* = 6). Except for the intact negative uteri, the caudal parts of the other uteri were submitted to traumatic endometrial curettage (Supplementary Figure 1).

**Figure 1:**
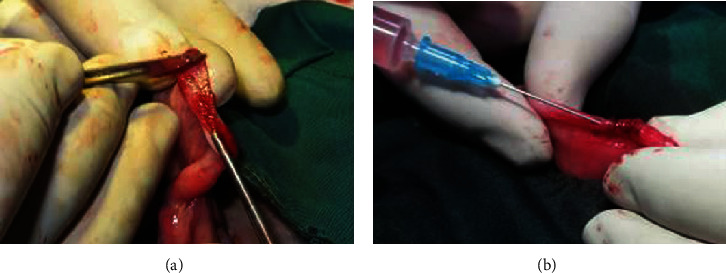
Surgical procedure of the evaluation of preventive effects of intrauterine stromal cell-conditioned media injection on uterine fibrosis after endometrial curettage in rabbits. (a) Intrauterine curettage of the rabbit uterus using a scalpel blade. (b) Injection of mesenchymal stromal cells or stromal cell-conditioned media after suturing of curettage uterus in treatment groups.

Briefly, the rabbits were anesthetized with a single intramuscular dose of a combination of ketamine 10% (35-40 mg/kg, Alfasan, Netherlands) and xylazine 2% (3-5 mg/kg, Alfasan, Netherlands) after preoperative overnight fasting. Following midline laparotomy incision, 5 cm long incisions were performed on the uteri. Incisions were located in the middle of each uterine tube. Using a scalpel blade through these incisions, the inside-out inverted endometrium was scratched (Figure 1(a)). The whole thickness of the endometrium was removed, and this was continued until bleeding was observed as an indicator of curettage completion. Then, uteri walls were sutured with 4-0 vicryl. The curettage positive control uteri were curettaged but not injected. Immediately after suturing of the uterus in both treatment groups, 1 mL of BM-MSCs (2 × 10^6^ cells) or BM-MSC-CM was injected into the uterus (Figure 1(b)). The intact negative control uteri were not curettaged or injected. Using 3-0 vicryl, abdominal muscles were sutured and 2-0 silk sutures closed the skin. Rabbits received flunixin meglumine (0.2 mg/kg, IM, Caspian Tamin™, Iran) immediately after the surgery and then after every 24 hours in 3 doses. Penstrep-400 (Nasr™, Iran, IM) was injected as an antimicrobial agent, just after the operation and continually for 3 days thereafter.

### 2.9. Histomorphometry of Uterus

The effects of both treatment techniques on the regeneration of the endometrium were evaluated 30 days after treatment by histomorphometric comparison with the controls. Rabbits were sacrificed with a high dose of pentobarbitone (1 g, Specia, France). Both uteri of rabbits were fixed for two weeks in 10% formalin buffer. After fixation, segments were embedded in paraffin, and 5 *μ*m thick sections were made from each block in the area of curettage with the help of suture landmarks.

They were stained with Masson's trichrome to be analyzed for histopathologic damages following curettage and the evolution of the regeneration process after treatment using morphologic indices and accumulation of fibrotic tissues in the curettage sites. The slides were visualized and photographed using a light microscope (CX21, Olympus, Japan) equipped with an adjusted digital camera (AM423U Eyepiece Camera, Dino-Eye, Taiwan). On the transverse sections, the endometrial area, the lumen area, and the total area of the uterus were measured using DinoCapture 2.0 software (Dino-Eye, Taiwan). The proportion of intact, damaged, and regenerated endometrial luminal epithelia was estimated by calculation of different indices. Histomorphometric indices according to the previously described method [14] included the lumen area/total horn area ratio (LA/THA), the endometrial area/total horn area ratio (EA/THA), the myometrial and perimetrial area/total horn area ratio (MPA/THA), the endometrial area/uterine wall area ratio (EA/UWA), and the myometrial and perimetrial area/uterine wall area ratio (MPA/UWA). After synechiae occurred, inflammatory elements and histopathologic changes were assessed in intact, curettaged, and treated uteri.

### 2.10. Masson's Trichrome Staining Protocol

The deparaffinized and rehydrated uterine sections were washed in distilled water. The slices were mordant in preheated Bouin's fluid overnight at room temperature. Then, they were stained in Weigert's iron hematoxylin solution for 10 min. The slides were washed under warm running tap water for 10 min and followed by a second rinse in distilled water. Using Biebrich's scarlet-acid fuchsin solution, slices were stained for 10 min and then rinsed in distilled water. Then, they were differentiated in phosphomolybdic-phosphotungstic acid solution for 10 min. The slides were frequently observed with the naked eye. Once collagen-rich areas lost their red color and became clear, the slides were transferred directly to an aniline blue solution to stain for 10 min. They were rinsed in distilled water and were differentiated in 1% acetic acid solution for 5 min. Slides were washed in distilled water and were very quickly dehydrated through 95% alcohol and then absolute alcohol to wipe off Biebrich's scarlet-acid fuchsin staining. Finally, the slices were made clear in xylene and mounted with resinous mounting medium [15].

### 2.11. Image Analysis of Fibrosis

**Figure 2:**
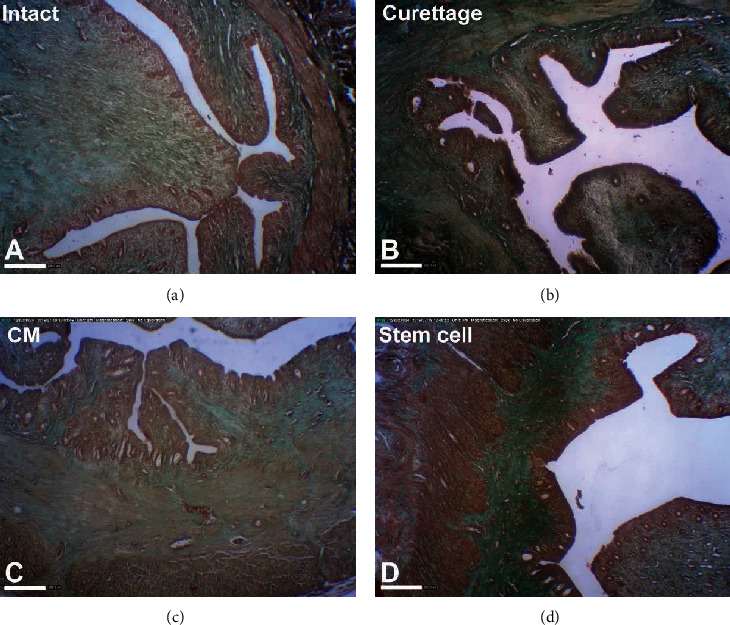
Histological evaluation of the preventive effects of intrauterine bone marrow-derived mesenchymal stromal cell- (BM-MSC-) conditioned media (CM) injection on uterine fibrosis after endometrial curettage in rabbits (Masson's trichrome staining). (a) The normal morphology of uteri was demonstrated in the intact group. (b) In the curettaged group, uteri were with moderate abnormal morphology and thinner endometrium after 30 days. (c) The treatment group after injection of BM-MSC-CM after 30 days. (d) The treatment group after injection of BM-MSC after 30 days (scale bars = 200 *μ*m).

Images from the regenerated and nonregenerated parts of uteri of experimental glass slides were obtained at the same resolution and magnification using a digital microscopic camera (AM423U Eyepiece Camera, Dino-Eye, Taiwan) and stored in a tiled Tiff format (Figure 2). Three separate fields (200 × 200 *μ*m^2^) of endometrial layers in each slide (*n* = 6) from different groups were cropped. Then, using the color threshold of ImageJ, the green and blue pixels which showed the presence of collagen fibers were selected, and the percentage of collagen areas were measured in each field [16].

### 2.12. Statistical Analysis

The normality test of the histomorphometric ratio indices was carried out by the Kolmogorov-Smirnov test. The mean and standard error (SE) ratios of LA/THA, EA/THA, MPA/THA, EA/UWA, and MPA/THA and the percentage of collagen in tissue fields were subjected to the Kolmogorov-Smirnov normal test, and data were analyzed by two-way ANOVA and LSD tests using SPSS 22 for windows (IBM SPSS Statistics for Windows, version 22, IBM Inc., Chicago, Illinois). *P* values less than 0.05 were considered statistically significant. Means and SE are reported in charts (GraphPad Prism version 5.01 for Windows, GraphPad Software Inc., San Diego, CA, USA).

## 3. Results

### 3.1. Rabbit BM-MSC Culture

**Figure 3:**
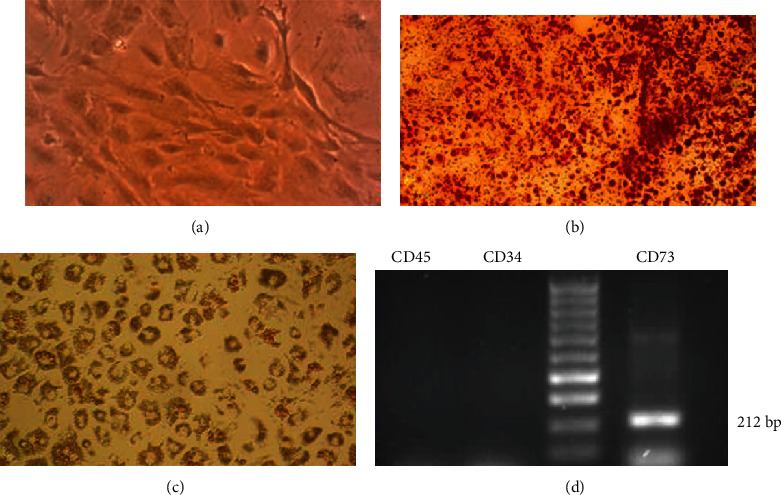
Characterization of rabbit bone marrow-derived mesenchymal stromal cells (BM-MSCs). (a) Fibroblast-like and spindle-shaped morphology of rabbit BM-MSCs, a typical characteristic of MSCs in passage 3 (×400). (b) BM-MSCs of rabbits cultivated in osteogenic medium and stained with Alizarin red (×100). (c) BM-MSCs of rabbits cultivated in adipogenic medium and stained with oil red O at day 21 after induction (×200). (d) Agarose gel electrophoresis of products of reverse transcriptase polymerase chain reaction (RT-PCR) of BM-MSCs revealing to be positive for CD73 (mesenchymal marker) and negative for CD34 and CD45 (hematopoietic markers).

In the primary culture, the cells had various forms. However, spindle cells or fibroblast-like cells as naturally occurring forms of BM-MSCs had the highest proportion among others, although flat or rounded cells were visible, too. Over time, the cells multiplied and gradually became denser. Thus, the cell colonies were detectable in a culture plate; these cell aggregations, in turn, attached to each other with the growth of cells, and consequently, the entire culture medium was loaded with cells after about two weeks. When the culture medium was filled with BM-MSCs, the cells were passaged using trypsin/EDTA. Rates of cell growth and proliferation were increased from this passage (which was called passage one) to the next passages; this continued in such a manner so that the cultivation surface was filled up with cells in over about three to four days. It should be noted that at this stage, the cells are often seen in the form of fibroblast-like cells or spindle cells (Figure 3(a)). This process continued until the third passage.

### 3.2. Rabbit BM-MSC Characterization

**Figure 4:**
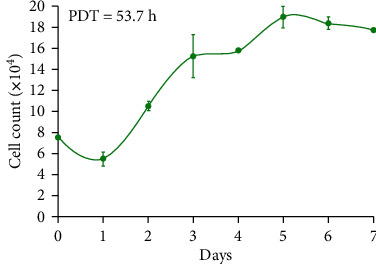
Mean and standard error of cell counts in growth curves of rabbit bone marrow-derived mesenchymal stromal cells, passage 3. PDT: population-doubling time. It should be noted that the samples are technical replicates.

For further confirmation of the BM-MSC characteristics in isolated cells, the osteogenic and adipogenic differentiation capacity of BM-MSCs were evaluated. After culture of BM-MSCs in osteogenic and adipogenic differentiation media separately, the cells were differentiated toward osteoblasts and also showed the presence of intracellular lipid droplets of adipocytes as, respectively, verified by positive staining with Alizarin red staining (Figure 3(b)) and oil red O staining (Figure 3(c)). To approve the expression of the surface marker of MSCs of rabbit on isolated BM-MSCs, the cells were analyzed using a RT-PCR assay.  Figure 3(d) displays positive expression for an MSC marker (CD73) and negative expression for hematopoietic stem cell markers (CD34 and CD45). According to our result, the PDT of passage 3 of the BM-MSCs was 53.7 h (Figure 4). BM-MSCs showed an acceptable proliferation rate in passage 3. In this study, the proliferation rate of BM-MSCs decreased gradually after 6 days.

### 3.3. Histopathologic Evaluation of Uterine Fibrosis Prevention

**Figure 5:**
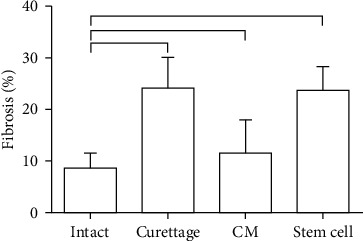
Mean and standard error of the amount of collagen fibers in the endometrial layer to compare the preventive effects of intrauterine bone marrow-derived mesenchymal stromal cell- (BM-MSCs-) conditioned media (CM) injection on uterine fibrosis after endometrial curettage in rabbit. The horizontal line above the columns show a statistically significant difference (*P* < 0.05).

The normal morphology of uteri was demonstrated in the intact group (Figure 2(a)). In the curettaged group, uteri presented with moderate abnormal morphology and thinner endometrium which were referred to as the model group. In all curettaged uteri, the histologic evidence of fibrosis was confirmed by Masson's trichrome staining (Figure 2(b)). Although the mean percentage of fibrotic tissue in the BM-MSC-CM group (Figure 2(c)) was lower than the curettaged and BM-MSC groups (Figure 2(d)), no significant difference in the percentage of fibrosis was observed among them (Figure 5). The confirmed fibrosis was about 25% of the endometrial layer in the curettaged group, which was higher than that in the intact group (Figure 5). There was no significant difference in fibrosis percentage between the BM-treatment groups and the control group (Figure 5). The mean percentage of fibrosis tissue of the BM-MSC-CM and BM-MSC groups was the same as the control group (Figure 5). Histological sections of all uteri have been presented in Supplementary Figure 2.

### 3.4. Histomorphometric Indices

**Figure 6:**
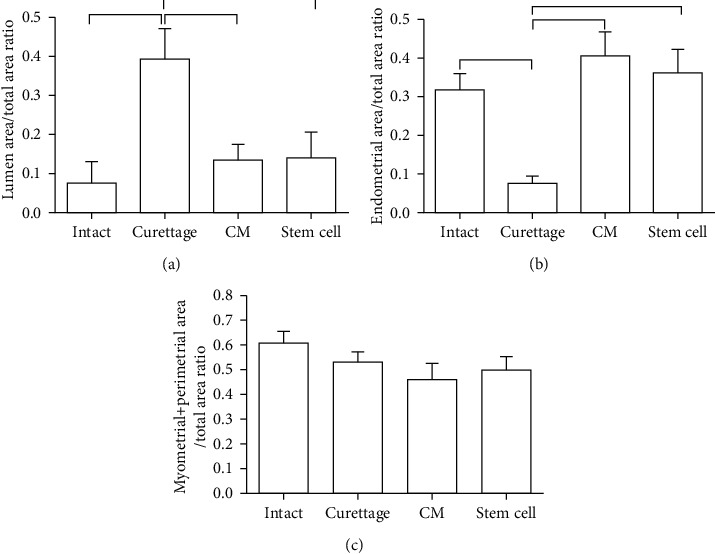
Mean and standard error of the uterine histomorphometric indices to compare the preventive effects of intrauterine bone marrow-derived mesenchymal stromal cell- (BM-MSC-) conditioned media (CM) injection on uterine fibrosis after endometrial curettage in rabbit. (a) Lumen area/total area ratio. (b) Endometrial area/total area ratio. (c) Myometrial + perimetrial area/total area ratio. The horizontal lines above the columns show a statistically significant difference (*P* < 0.05).

Evaluation of histomorphometric indices including the LA/THA ratio, the EA/THA ratio, and the MPA/THA ratio showed that endometrial layers in the curettaged and the treated groups had grown more than the control group after 30 days (*P* < 0.05; Figure 6). The curettaged, BM-MSC-CM treatment, and BM-MSC treatment groups had significantly lower means of the lumen area/total area ratio than the intact control group (*P* < 0.05; Figure 6(a)). Moreover, the curettaged, BM-MSC-CM treatment, and BM-MSC treatment groups had significantly higher means of the EA/THA ratio than the intact control group (*P* < 0.05; Figure 6(b)). Comparison of the mean of the MPA/THA ratio showed that there were no significant differences among the four groups (*P* > 0.05; Figure 6(c)).

**Figure 7:**
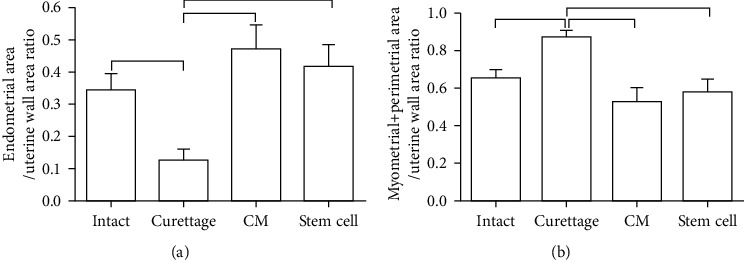
Mean and standard error of the uterine histomorphometric indices to compare the preventive effects of intrauterine bone marrow-derived mesenchymal stromal cell- (BM-MSC-) conditioned media (CM) injection on uterine fibrosis after endometrial curettage in rabbit. (a) Endometrial area/uterine wall area ratio. (b) Myometrial + perimetrial area/uterine wall area ratio. The horizontal line above the columns show a statistically significant difference (*P* < 0.05).

Evaluation of histomorphometric indices including the EA/UWA ratio and the MPA/UWA ratio showed that endometrial layers in the curettaged and treated groups had grown more than the control group after 30 days (*P* < 0.05; Figure 7). The curettaged, BM-MSC-CM treatment, and BM-MSC treatment groups had significantly higher means of the endometrial area/uterine wall area ratio than the intact control group (*P* < 0.05; Figure 7(a)). Moreover, the curettaged, BM-MSC-CM treatment, and BM-MSC treatment groups had significantly higher means of the myometrial + perimetrial area/uterine wall area ratio than the intact control group (*P* < 0.05; Figure 6(b)).

## 4. Discussion

Injection of BM-MSC-CM immediately after curettage reduced the formation of fibrotic tissues in rabbit uterus but did not have any effect on uterine wall thickness. In addition, injection of BM-MSC-CM had a slightly higher preventive effect than BM-MSC injection on the formation of fibrotic tissues. Consistent with our findings, Liu et al. [5] and Ho et al. [4] using a full uterine punch model showed the therapeutic effect of MSC-CM for uterine fibrosis. CM contains exosomes which are secreted from the cells [17] and can induce tissue regeneration [18, 19] or cell differentiation [20, 21]. Bone marrow-derived-, [22], uterine-derived-, [23], and adipose-derived- [24] MSCs' exosomes also restored endometrial function in a rat model of adhesions. These exosomes contain miRNAs which induce tissue regeneration [25].

Several different growth factors and cytokines bearing therapeutic properties are released from stem cells into their CM, which play key roles in angiogenesis as well as in the regeneration of damaged tissues and organs [26]. They include vascular endothelial-derived growth factor (VEGF), platelet-derived growth factor (PDGF), epidermal growth factor (EGF), insulin-like growth factor I (IGF-I), insulin-like growth factor II (IGF-II), hepatocyte growth factor (HGF), and fibroblast growth factor 2/basic fibroblast growth factor (FGF-2/bFGF) [27].

In a study by Xiao et al. [28], the positive effect of miR-340 on the function of the cell therapy of Asherman's syndrome was confirmed. Furthermore, consistent with our findings, comparing BM-MSC-CM and MSC-CM regarding the time of regeneration, MSC exosomal treatment restored the damage of Asherman's syndrome in tissue over a shorter time period than the MSCs [23]. Therefore, injecting BM-MSC-CM can be used for the prevention of uterine fibrosis provided that it is injected immediately after curettage.

Allotransplantation of BM-MSCs immediately after curettage reduced the formation of fibrotic tissues in rabbit uterus but did not have any effect on uterine wall thickness. Furthermore, BMDC injection in uteri of the cellular proliferation-suppressed mice resulted in an increase in endometrial thickness [3, 29]. [30]. As BM contains both hematopoietic and mesenchymal cells, two groups of studies showed the effect of hematopoietic stem cells (HSCs) and MSCs on the induction of uterine regeneration. HSC injection induced an increase in the thickness of a thin endometrium or formation of endometrial blood vessels [31–33]. Beyond animal models, BMDCs and HSCs also cured Asherman's syndrome in humans [34–36].

Besides, since uterine epithelium tissue contains endometrial MSCs [37–40], the role of transplanted MSCs in the prevention of uterine tissue fibrosis sounds plausible. However, during uterine injury, endometrial MSCs and their microenvironments are disturbed, resulting in the reduced capability of the uterus in tissue regeneration. Therefore, fibrotic tissue as a pathological healing process replaces normal endometrial tissue. In the current study, BM-MSCs were injected immediately after curettage to prevent fibrotic tissue formation. To the best of our knowledge, this method of HSC marker cell therapy for the prevention of fibrosis has not been investigated so far. However, in previous studies, the treatment of uterine injury after fibrotic tissue formation showed that MSC injection improved regeneration of thin endometrium in mouse [41, 42] and rat [43, 44] models. For endometrial regeneration, various sources of MSCs have been used including allotransplantation of BM-MSCs [13], endometrial MSCs [42], and adipose MSCs [24, 44] or xenotransplantation of human amniotic MSCs [45]. Therefore, the findings of our study in combination with previous findings of uterine fibrosis treatment confirm the preventive effects of BM-MSCs on uterine fibrosis.

In our study, for the first time, the rabbit model of uterine fibrosis has been used for the treatment of uterine fibrosis induced by uterine curettage. The methods through which uterine damage are induced vary from mechanical [41] to chemical [42] damages. Mechanical methods such as using a uterus punch [4, 5] is not similar to human endometrial curettage. However, different studies have used various species as models for uterine fibrosis induction in order to investigate MSC therapy including mice [41], rat [43, 44], and rabbit, which might be more beneficial than others. In the present study, we used the rabbit model due to the larger size of its uterus compared to murine models which provide a more controllable condition for investigating endometrial damage. Furthermore, the timing of estrous cycles in murines is 4 to 5 days and hormonal alterations of shorter estrus cycles can reduce the comparability of these models to human uterine tissue regeneration. Rabbits have a longer diestrus phase than murines, and the uterine fibrosis model of rabbits [14, 46] seems to be more similar to the human model than the murine models.

Considering the fact that development of an applied method for the prevention of uterine fibrosis using cell therapy methods requires a practical protocol in emergency situations, intrauterine exosome injection as an expensive protocol or CM injection immediately after curettage as a cheap solution can be suggested. Allotransplantation injection of CM may allow the operator to perform the therapeutic procedure in standard conditions during the appropriate time period. However, human clinical trials are necessary to confirm it.

## 5. Conclusions

Based on the findings of the current study, injecting BM-MSC-CM immediately after curettage has the same effective role in preventing and reducing uterine fibrosis as injecting BM-MSC in a rabbit model. Therefore, the stressful condition of cell therapy and its possible complications can be replaced by the CM injection method in the prevention of uterine fibrosis.

## Figures and Tables

**Table 1:** Sequences of RT-PCR primers used to quantify the expression of bone marrow-derived mesenchymal stromal cell-specific surface markers (CD73) and hematopoietic stem cell-specific surface markers (CD45 and CD34) in rabbit.

Primer	Primer sequence	Amplicon length (bp)
CD34-F CD34-R	ACCATCTCAGAGACTAGAGTC GAAAGTTCTGTTCTGTTGGC	512
CD45-F CD45-R	CAGTACTCTGCCTCCCGTTG TACTGCTGAGTGTCTGCGTG	269
CD73-F CD73-R	TACACCGGCAATCCACCTTC CTTGGGTCTTCGGGAATGCT	212

## Data Availability

The data used to support the findings of this study are available from the corresponding author upon request.

## Abbreviations

BM-MSCs: Bone marrow-derived mesenchymal stromal cells
cDNA: Complementary DNA
CM: Conditioned media
DMEM: Dulbecco's Modified Eagle's Medium
DMSO: Dimethyl sulfoxide
EA/THA: Endometrial area/total horn area ratio;
EA/UWA: Endometrial area/uterine wall area ratio
FBS: Fetal bovine serum
HSCs: Hematopoietic stem cells
LA/THA: Lumen area/total horn area ratio
MPA/THA: Myometrial and perimetrial area/total horn area ratio
MPA/UWA: Myometrial and perimetrial area/uterine wall area ratio
MSCs: Mesenchymal stromal cells
PBS: Phosphate-buffered saline
PDT: Population-doubling time
RT-PCR: Reverse transcription-polymerase chain reaction.

## Supplementary Materials

Supplementary Materials Supplementary Figure 1: the study design for the 12 rabbits indicating the treatment of 2 uterine horns in each rabbit. CM: conditioned media. Supplementary Figure 2: histological evaluation of the preventive effects of intrauterine bone marrow-derived mesenchymal stromal cell- (BM-MSC-) conditioned media (CM) injection on uterine fibrosis after endometrial curettage in rabbits of all groups (Masson's trichrome staining).
