# Supplementary material for: Preventive Effects of Intrauterine Injection of Bone Marrow-Derived Mesenchymal Stromal Cell-Conditioned Media on Uterine Fibrosis Immediately after Endometrial Curettage in Rabbit
Source: Stem Cells Int. 2020 Nov 7;2020:8849537. doi: 10.1155/2020/8849537 (PMC7666625; doi:10.1155/2020/8849537)
Supplement: Supplementary Materials — Supplementary Figure 1: the study design for the 12 rabbits indicating the treatment of 2 uterine horns in each rabbit. CM: conditioned media. Supplementary Figure 2: histological evaluation of the preventive effects of intrauterine bone marrow-derived mesenchymal stromal cell- (BM-MSC-) conditioned media (CM) injection on uterine fibrosis after endometrial curettage in rabbits of all groups (Masson's trichrome staining). [file 8849537.f1.docx]

# Supplementary Figures


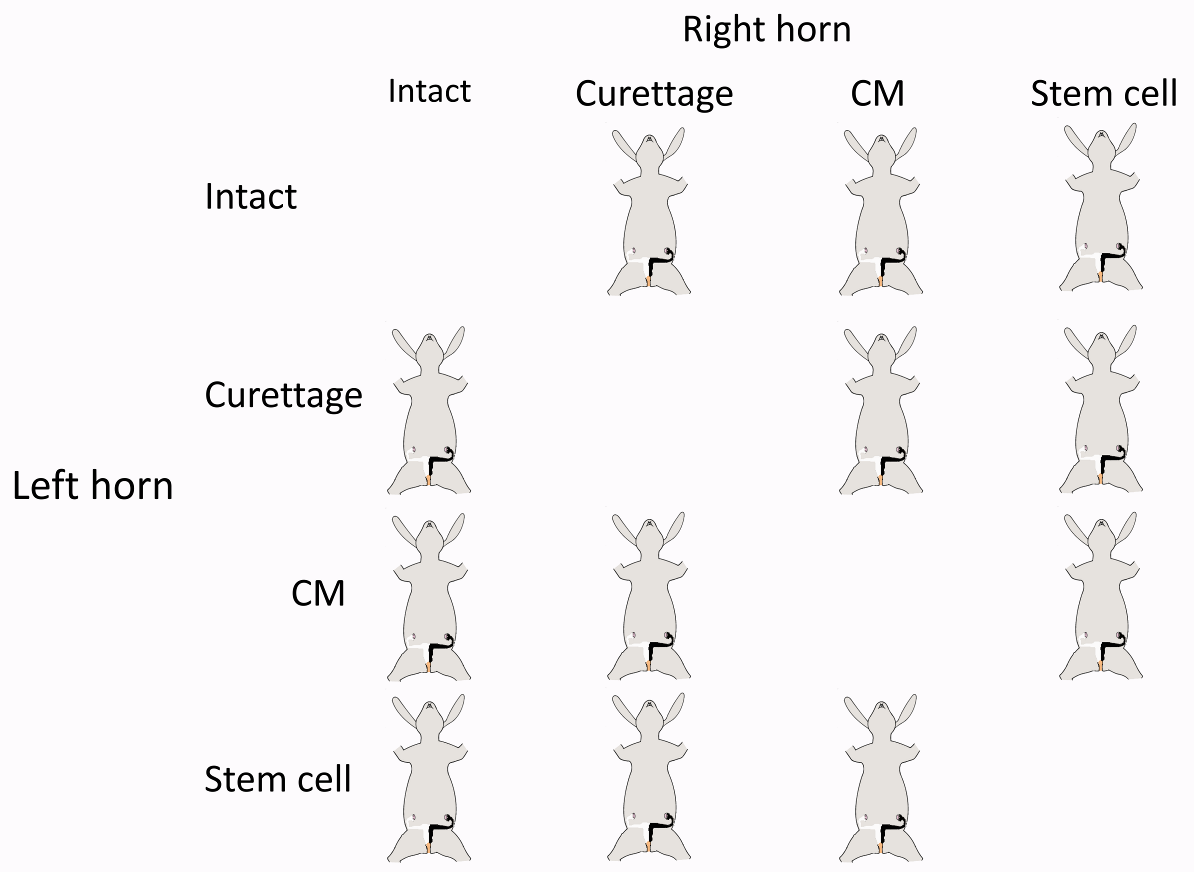


Supplementary Figure 1. The study design for the 12 rabbits and indicate the treatment of 2 uterine horns in each rabbit. CM, conditioned media.


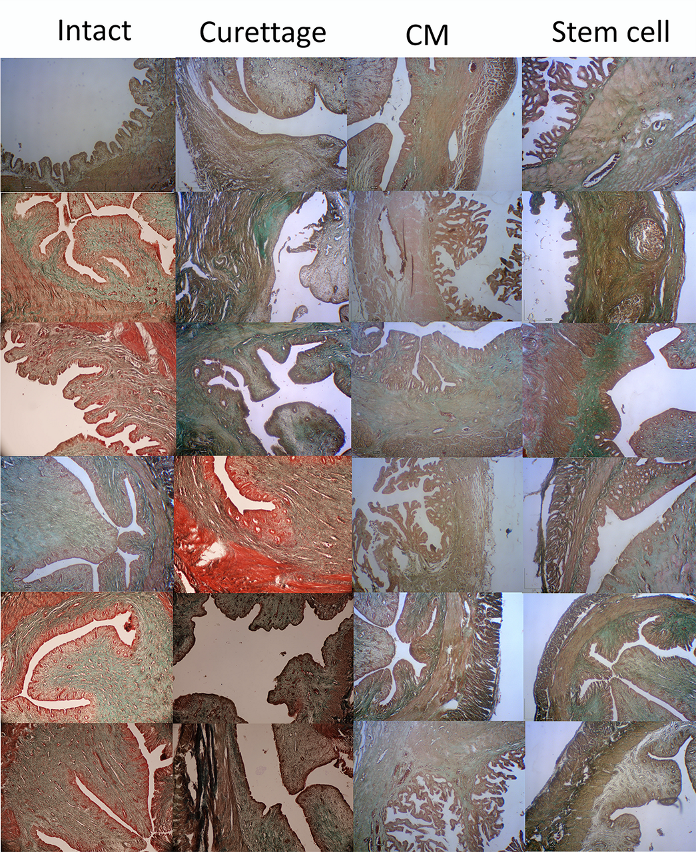


Supplementary Figure 2. Histological evaluation of the preventive effects of intrauterine bone marrow-derived mesenchymal stromal cell (BM-MSCs)-conditioned media (CM) injection on uterine fibrosis after endometrial curettage in rabbit of all groups (Masson’s trichrome staining).
